# Characterization of Waste *Nicotiana rustica* L. (Tobacco) Fiber Having a Potential in Textile and Composite Applications

**DOI:** 10.3390/polym16081117

**Published:** 2024-04-17

**Authors:** Sabih Ovalı

**Affiliations:** Department of Textile Engineering, Adıyaman University, 02040 Adıyaman, Turkey; sovali@adiyaman.edu.tr

**Keywords:** *Nicotiana rustica* L., waste fiber, textile, sustainability, composite

## Abstract

*Nicotiana rustica* L. (NRL) is a type of tobacco plant, and its stalk waste is a potential lignocellulosic source for obtaining cellulose fibers freely available in nature. However, they are left in fields after harvesting, and this study provides a green and sustainable method to reuse tobacco waste. Fiber was obtained by retting the plant stalks in water and decomposing them naturally in three weeks. NRL fiber was characterized by comparing it with known bast fibers, and tests were applied to examine its physical, chemical, mechanical, morphological, and thermal properties. With its high cellulose content (56.6 wt%), NRL fiber had a high tensile strength (113.4 MPa) and a good crystallinity index (70%) that helped it to bond with other fibers in the composite matrix. Furthermore, the fiber is an environmentally friendly alternative to synthetic fibers with a diameter of 36.88 μm and low density (1.5 g/cm^3^). The NRL fiber was found to have a semi-crystalline structure and large crystalline size, which makes it hydrophobic. The thermal gravimetric analysis showed that it can be durable (353.9 °C) in higher temperatures than the polymerization temperature. As a result, it can be concluded that NRL fiber has the potential to be used as a reinforcement in polymer composites, technical textiles, and agricultural applications.

## 1. Introduction

*Nicotiana rustica* L. (tobacco) is a tall and leafy plant growing annually and is a member of the Solanaceae family, which are aromatic and industrial plants. Cigarettes, pipes, hookahs, cigars, snuff, chewing tobacco, and cigarettes were among the forms of tobacco consumed in Europe after it was first used as a medicine [1,2,3]. Tobacco is one of the agricultural products that can adapt to various geographical conditions to the fastest and most reasonable extent [4]. Tobacco plants are adaptable to all climate conditions, can grow in small-scale operations, and adapt to poor soils for production [1]. Due to the climate conditions, NRL is primarily grown in Turkey’s Eastern and Southeast Anatolia regions, and it is the most widely grown tobacco species after *Nicotiana tabacum* L. (NTL) [4,5].

Large amounts of biomass are typically produced during tobacco harvesting [6,7]; these are post-harvest waste materials, leftover aboveground components like leaves and stalks. According to estimations, tobacco production generates more than 200 million tons of waste yearly [8]. It is typically buried to add organic matter to the soil and dispose of waste materials that seem worthless. However, contamination could arise from the burial of crop residual biomass, which has high toxicity to human health and the natural environment [9,10].

Several research papers are on utilizing tobacco wastes to extract chemicals such as nicotine, cellulose, and protein in pharmaceuticals, agriculture, and other industries [11,12,13,14,15].

However, the current extraction methods have high energy and low extraction efficiency; besides, they are very complicated and cause water and air pollution. Thus, it is essential to utilize tobacco waste with green treatment methods with effective and eco-friendly applications, such as chemically converting biomass into textile fibers. Agricultural waste and crop residues have lignocellulosic composition and are structured with functional groups [16]. Thus, they have high surface functionality, help with yarn twisting, and can interact with other fibers in composite production. It has been widely reported that various agricultural biomasses such as hemp [17], jute, and flax [18] are potential sources for fiber production and use in various textile applications due to their high cellulose and lignin content.

In determining the cellulose content of large tobacco plants, the stalks and leaves provide the necessary data to processors engaged in biomass processing [19]. Tobacco stalk residues can contain up to 40% cellulose by weight [20]. Cellulose is the primary building block of plants [21] and the most abundant biopolymer in nature [22,23,24]. Cellulose is the primary component in natural fibers and constitutes more than 30–40% of the total biomass [25]. Cellulose is the strongest and stiffest part of natural fiber [26]. The growing rate of textile fibers is 3.1% per year; furthermore, they were in huge demand (75.5 million tons) in 2010, and it is predicted that this demand will be 133.5 million tons by 2030 [25]. Cellulose is significant for the textile industry; fortunately, it is readily available in nature, making it a renewable resource.

There are several studies on the fiber production of tobacco biomass, mainly on the characterization of tobacco stalks for composite [27,28], pharmaceutical [29], agriculture [30], bioenergy [31], and cosmetic [32,33] applications.

Extracting cellulose-based fibers from agricultural wastes can be analyzed in three categories due to their methods as physical, chemical, and enzymatic applications [34]. Physical methods, like mechanical fibrillation [29] and mechanical grinding [35], are generally chosen to obtain cellulose nanofibrils; however, they are time and energy-consuming. Additionally, the crushing process decreases the crystallinity of cellulose and increases the polymer solubility, which is not desired. Mechanical and chemical methods are combined to save energy in some applications like ultrasonication [36] and steam explosion [37]. They help to eliminate lignin and hemicelluloses and leave cellulose intact. In chemical methods, strong acids are used to hydrolyze and remove the amorphous regions in cellulose fibers [38]. Hydrochloric and sulfuric acids are mainly strong acids; they can react with the hydroxyl groups on the fiber surface and allow the anionic sulfate ester groups to graft. In addition, cellulose fibers are partially separated with alkaline hydrolysis; this helps to improve their physical and chemical characteristics [39]. However, these strong chemical treatments are not environmentally and economically friendly. Thus, enzymatic methods have environmental benefits; in the cellulosic fibers, the restrictive and selective hydrolysis of cellulose and other components can occur [40]. However, although the enzymatic methods are more efficient, easier to operate, and have lower energy costs than chemical methods, they are still not preferred due to economic and technical problems.

About 410,000 tons of biomass is generated annually in Turkey because of tobacco harvesting, often left in the fields [41]. Though the reported literature has studied obtaining fiber from NTL plant stalks using different methods, this study focuses on obtaining cellulosic fiber by retting the NRL plant stalks in water and waiting for them to decompose naturally as an environmentally friendly application. The fiber was isolated from the tobacco plant stalks using a physical (mechanical fibrillation) method after decomposition. The morphological, chemical, physical, mechanical, and thermal characterization of the NRL fiber was investigated to utilize this high amount of tobacco stalks and evaluate the potential of NRL plants to supply fiber in the textile and composite industries for agricultural, industrial, and automotive applications. Tobacco stalks have shorter growing times and lower lignin; thus, they are a sustainable option for cellulose-based fiber.

## 2. Materials and Methods

### 2.1. Fiber Extraction

All the fiber studied was obtained from tobacco stalks (*Nicotiana rustica* L.) taken from Tekpınar village in Adıyaman, southern Turkey. The tobacco leaves grown there for the cigarette industry were harvested in three stages according to yellowing color. Tobacco stalks were collected towards the end of October 2023 after the third stage of tobacco leaves was harvested. All the tobacco plants were taken from one harvest to keep variations at a minimum.

**Figure 1:**
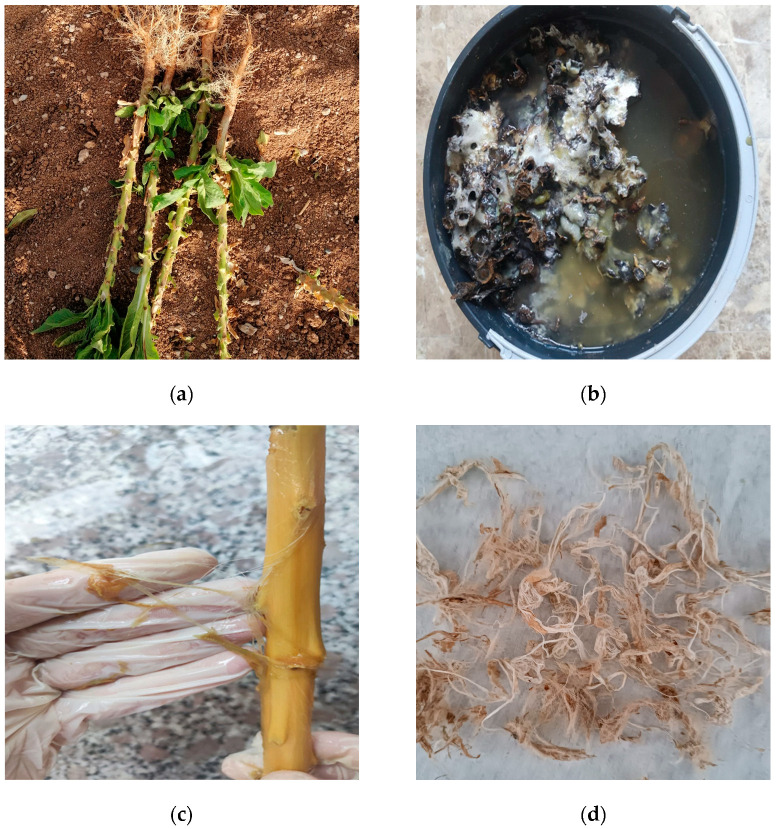
Fiber extraction from NRL plant stalk waste. (**a**) Waste NRL plant stalks, (**b**) NRL plant stalks retting in water, (**c**) fibers removed from stalks and washed with distilled water, and (**d**) extracted dry NRL fibers.

Fiber extraction with the water retting method is shown step by step in Figure 1 [42,43,44]. After harvesting, the waste tobacco stalks were collected (Figure 1a), then roughly cleaned, cut into pieces to fit into the bucket, and placed in the bucket filled with water (Figure 1b).

The NRL stalks were left to decompose for about three weeks by adding only water to the bucket. The water penetrated the center of the NRL stalks, swelled the cells inside, and burst the layer on the outer part of the fiber.

The moisture increased bacteria, producing decay so that the cellular textures and gummy substances dissolved and rotted away on NRL stalks. Afterward, the NRL fibers began to separate and were washed to extract them from the stalks (Figure 1c). Finally, the fibers were left to dry for two days (Figure 1d). The obtained fibers were subjected to relevant tests performed after drying.

### 2.2. Physical Analysis

The density measurement of the extracted fibers was performed according to the ASTM D8171-18 standard [45], which is also widely used in the literature. This method measures the buoyancy created by a saturated fiber submerged in a fluid using Archimedes’ principle. The mean fiber length was ascertained by choosing 100 fibers at random from a tuft of NRL fiber sample. After straightening each fiber sample, a measuring scale was used to determine its length. The standard deviation (SD), mean, and coefficient of variation (CV) were calculated using the obtained fiber length.

### 2.3. Chemical Composition Quantification

The main chemical composition (cellulose, lignin, and moisture) of NRL fiber was determined after removing the excess moisture from the sample fibers.

One gram of NRL sample and 1.72% sodium chlorite solution (Sigma-Aldrich, St. Louis, MO, USA, CAS: 7758-19-2) were added to a sterile dry container. The sample was acidified by adding a few drops of strong sulfuric acid. The solution refluxed for one hour. Subsequently, the suction method was used to drain the excess fluid. The resulting mixture was treated with ammonia before being rinsed with distilled water. The left fibers were dried and then weighed. Then, the cellulose ratio was determined using Equation (1):

C (%) = (R/D) × 100(1)

where D is the dry weight of samples (g), R is the weight of left fibers (g), and C is the cellulose ratio.

One gram of fiber was dried for four hours at 100 °C in the oven [46], after which it was cured for one h at room temperature with a 4% NaOH (Merck, Darmstadt, Germany, CAS: 1310-73-2) solution and 5% HCl (Sigma-Aldrich, CAS: 7647-01-0, 36.5–38%). Following one hour of processing in an oven, the HCl was extracted from the sample using suction. The amount of hemicellulose was found by subtracting the end weight from the initial weight.

One gram of NRL fiber was soaked in 12.5 mL sulfuric acid for one hour with 72% concentration (Sigma-Aldrich, CAS: 7664-93-9, 95–97%) at room temperature. Afterward, it was mixed with 300 mL of distilled water and stood for two hours. The solvents were carefully drained, and then, finally, the silt was weighed, and it was concluded that the amount of silt represented the amount of lignin.

### 2.4. Categorization of Chemical Functional Groups in NRL

A Perkin Elmer Spectrum BX Fourier Transform Infrared Spectrometer was used to obtain Fourier-transform infrared (FTIR) spectra of NRL fibers. A signal-to-noise ratio resolution of 2 cm^−1^ with 32 scans/minute was used to record the data in the range 4000–400 cm^−1^. FTIR analysis was used to obtain the functional groups of NRL fiber.

### 2.5. X-ray Photoelectron Spectroscopy Analysis (XPS)

The NRL fiber’s elemental composition was determined using a monochromatic Al-Ka X-ray source (Thermo Fisher Scientific Inc., East Grinstead, UK) with 1486.7 eV energy and a focused beam with a diameter of 300 µm. For XPS studies, a 50 eV pass energy was employed, yielding a 0.1 eV resolution over an energy of 1361 and 10 eV. Following ionic Ar gas sputtering, data were gathered by executing 10 scans from a single site before surface analysis.

### 2.6. XRD Analysis and Crystallinity Index Determination

A standard tool for quantifying the amount of crystalline fraction in cellulosic materials and measuring how much they have changed after undergoing various physicochemical and biological treatments is the crystallinity index (CI) [24]. The crystalline phase content, or CI, is just the present percentage. Consequently, determining cellulose structure depends heavily on measuring CI. NRL fibers’ crystalline structure was determined with Segal’s equation in Equation (2) [47]. The total of the crystalline and amorphous components was represented by the height of the 200 peaks between the scattered angles of 2θ = 22° and 23°. The amorphous-only component was represented by the empirical method’s minimum intensity of roughly 18° between the overlapped peaks and 200.

(2) $$CI\left(\%\right)=\frac{I_{2\;0\;0}-I_{am}}{I_{2\;0\;0}}\times 100$$

where I_am_ stands for the 2θ intensity at roughly 18°, which is the low value between the overlapped peaks (1 $\bar{1}$ 0 and 1 1 0) and (2 0 0) peak. I_2 0 0_ is associated with the (2 0 0) lattice plane between 22° and 23°, showing the peak at maximum intensity [47].

The Rigaku miniflex600 (Rigaku Corp., Tokyo, Japan) device was used to determine the crystalline structure of the NRL fibers, and the reflection of the fibers’ X-ray diffraction (XRD) generated the data pattern. The crystallinity index (CI) was calculated using this data. The fibers were ground up, and the moisture was extracted for 24 h at 105 °C before the measurement procedure. The device worked at 40 kV using 36 mA current, with the source of the X-ray being Cu-Kα radiation (λ-Kα = 1.54 Å). The analyses took place over fifteen minutes, with each sample being scanned at a width of 2θ = 5–55 degrees.

### 2.7. Investigation of the Thermal Degradation Behavior

To assess thermal stability, TG and DTA measurements of NRL fibers were performed using the Seiko SII TG/DTA 7200 (Seiko, Chiba, Japan) instrument. We heated 5 mg fiber specimens to determine the chemical stabilization characteristic of the fibers at a rate of 10 °C/min at 30–700 °C in an environment of nitrogen.

### 2.8. Examination of Surface Morphology

Longitudinal and cross-sectional images of NRL fibers were captured using an SEM-Zeiss/Evo LS10 (Jena, Germany) device. The fibers were coated with a conductive gold film using sputter coating to prevent the charging effect. All characterizations regarding the structure of the surface, porosity of the surface, and fiber diameter were performed accordingly.

### 2.9. Single Fiber Test

The tensile tests of NRL fibers were made on 10 mm long fibers using an Instron Universal Tester with a 50 N load cell and a 0.1 mm/min speed. According to ASTM D 3822-07 standard [48], the tests were conducted under standard atmospheric conditions. For the single fiber test, 30 repetitive tests were performed due to the irregular shape commonly found in natural fibers [49].

### 2.10. Fiber Yield Analysis

Ten-gram NRL stalk samples were weighed, and fiber samples were obtained from these stalks. The sample fibers were put in an oven and kept at 110 °C for 12 h. Then, the dried fiber samples were taken out of the oven and weighed. The fiber yield was determined using Equation (3) [15].

Fiber yield (%) = (oven dried fiber/sun dried NRL stalks) × 100(3)

### 2.11. Moisture Content

NRL fibers’ moisture content varies due to the environmental conditions where the NRL stalks are collected. The NRL fibers were dried in an oven, and their moisture content was calculated with Equation (4) [15].

Moisture content (%) = ((wet fiber mass − dry fiber mass)/wet fiber mass) × 100(4)

## 3. Results and Discussion

### 3.1. Physical and Chemical Properties of NRL Fiber

**Figure 2:**
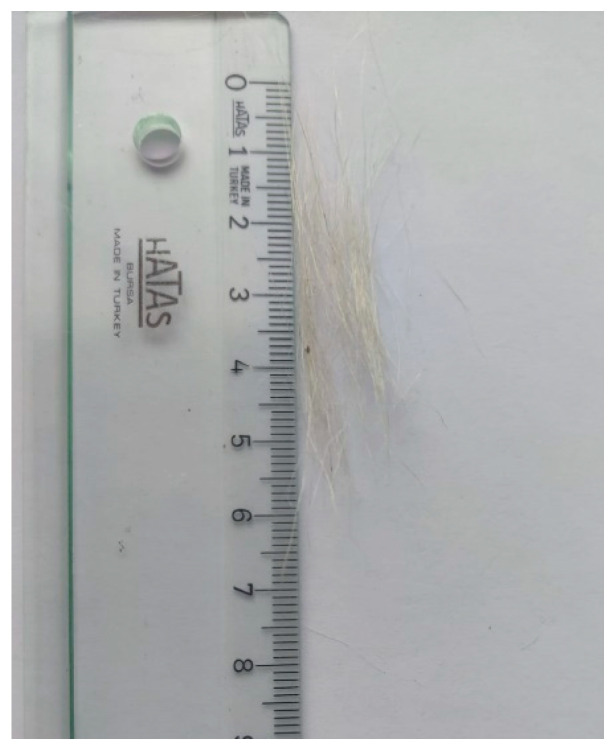
Combed NRL fibers.

Fiber length is one of textile fiber’s crucial properties for its spinnability. With an average length of 30.2 mm, NRL fibers ranged from 16 to 55 mm. Compared with cotton fibers, NRL fibers had a similar average length, as shown in Table 1. The distribution of fiber lengths for 100 randomly chosen NRL fibers is shown in Figure 2. The measured fibers had a standard deviation of 10.2 mm and a CV of 31.41%. NRL fiber was similar to bast fibers due to its form. Thus, it could be tested to see if it applies to yarn spinning as cotton fiber. When the form of NRL fiber was considered as bast fiber and compared with other bast fibers, its length was similar to that of hemp but much shorter than others.

The density of NRL fiber was calculated as 1.50 g/cm^3^. It was higher than sisal fiber and similar to cotton and other bast fibers in the literature [50,51]. Its average diameter and density were similar to all bast fibers, making it convenient for composite production as a reinforcement material [52].

**Table 1:** Comparison of physical properties for NRL and other bast fibers.

Fiber	Length (mm)	Diameter (μm)	Density (g/cm^3^)	Ref.
*Nicotiana rustica* L.	16–55	26–46	1.50	In this study
Cotton	10–50	14–21	1.52–1.56	[50,51]
Hemp	15–25	15–30	1.48–1.49	[50]
Flax	Up to 900	17–20	1.50	[50,51]
Jute	Up to 4000	14–20	1.44–1.49	[50]
Sisal	800–1200	7–47	1.20–1.22	[50]
Ramie	Up to 1900	40–60	1.51–1.55	[50,51]

The mechanical properties and the degradation of the fiber in nature are affected by its chemical content. At the same time, the existing chemical ratios of cellulosic fibers, the fiber extraction method and growing weather conditions, the way they are harvested, the test method applied, and the experience of the person applying the analysis cause variability in the analysis results [53].

Although tobacco is grown for its leaves to use in cigarette production or the pharma industry, its stalks are similar to bast crops primarily used in textile and composite production. Table 2 shows the chemical contents of NRL compared with other bast fibers. NRL fiber cellulose content was obtained as 56.6%, close to kenaf fiber but lower than others. Hemicellulose causes damage to the microfibrils of the fiber, negatively affecting the strength. NRL fiber has lower hemicellulose content (11.8%) than other bast fibers. The researchers found the hemicellulose content of NTL was 14.33% [54] and 22.8% [55]; thus, the NRL fibers had lower hemicellulose content than other types of tobacco plants, which affected their tensile strength positively.

Lignin provides affinity due to its amorphous properties and the presence of hydroxyl groups. Still, it causes fractures during processing due to its hardness and causes negativities in the end-use [56]. The lignin content was found to be 14.97% for NRL fiber; this is higher than that of other bast fibers and close to that of NTL fiber’s lignin content (18.90%) found in the research [57].

Stalk fibers are found in the outer bark of plants in bundles adhered with pectin. The digestion method separated the fibers from pectin to obtain individual fibers. Fibers can be obtained from the plant stalks using water or dew digestion or chemical applications. It is thought that pectin is primarily separated from NRL fiber extracted by the water digestion method [58,59]. Therefore, the pectin content of NRL fiber could not be determined.

The fiber yield of NRL fiber was calculated with Equation (3) as 51%; the researchers [15] found the yield of NTL was 50–70%, showing that the calculated NRL fiber yield was average. Moreover, the moisture content of NRL fiber was determined to be 13.15% with Equation (4). Similar moisture content was determined with hemp, flax, and jute fibers.

In addition, the researchers [15] found the moisture content of NTL was 8.36–10.56%, which was close to the calculated NRL fiber moisture content, as shown in Table 2.

**Table 2:** Chemical contents of NRL and other bast fibers.

Fiber	Cellulose (%)	Hemicellulose (%)	Lignin (%)	Moisture Uptake	Ref.
*Nicotiana rustica* L.	56.6	11.8	14.97	13.15	In this study
Hemp	67–75	16–18	3–5	6.2–12	[42]
Flax	62–71	16–18	3–4.5	8–12	[42]
Jute	59–71	12–13	11.8–12.9	12.5–13.7	[42]
Kenaf	45–57	21.5	12–13	6–12	[60]
Ramie	68–76	13–14	0.6–2	7.5–17	[60]

### 3.2. Mechanical Properties of NRL Fiber

The cchemical structure of cellulosic-based textile fibers (cellulose, hemicellulose, and lignin) affects their mechanical properties [61]. The mechanical properties of NRL and other natural fibers are given in Table 3.

As a result of the single fiber tensile analysis of NRL fiber, the tensile strength (113.40 MPa), tensile modulus (2.87 GPa), and elongation (3.94%) were determined. The lower tensile strength value of NRL fiber compared to other bast fibers is related to the lower cellulose ratio [61]. Studies show that strength increases with an increasing cellulose ratio [63]. Non-cellulosic ingredients in the chemical structure of the fiber may cause low fiber strength [64].

### 3.3. Surface Morphology of NRL Fiber

**Figure 3:**
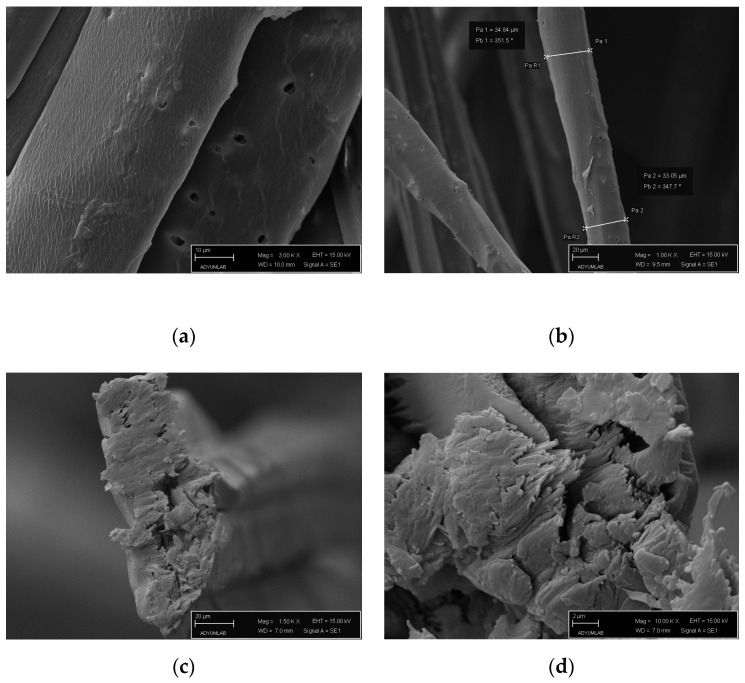
SEM images of NRL fibers: (**a**,**b**) longitudinal, and (**c**,**d**) cross-sectional images.

Figure 3 shows the cross-sectional and longitudinal SEM images of NRL fibers obtained at 100×, 300×, and 1000× magnification.

The fiber diameter was approximately 36.88 ± 10.54 μm, averaging ten measurements from different longitudinal section images. The images in Figure 3a,b show pores and impurities on the fiber’s surface [65]. The NRL fiber surface structure was similar to other bast fibers in the literature. Chemical surface treatment should be applied to increase the surface roughness before NRL fiber-reinforced composite applications.

### 3.4. FTIR Analysis

**Figure 4:**
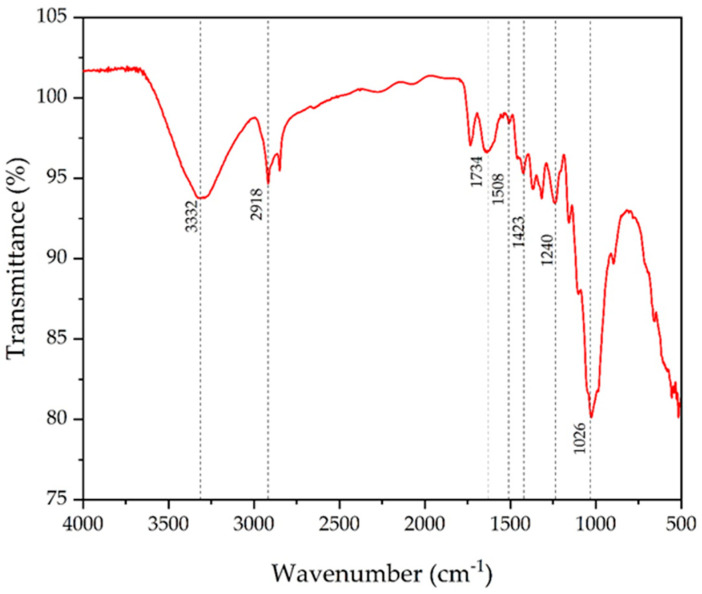
FTIR spectrum of NRL fiber.

The functional groups of NRL fiber are shown in the FTIR analysis curve in Figure 4. The peak at 3332 cm^−1^ was formed by -OH stretching, and the peak at 2918 cm^−1^ was formed by CH stretching; these indicate the presence of cellulose [66,67]. Additionally, the peak at 1734 cm^−1^ was due to the C=O stretching of hemicellulose [68], and the peak at 1508 cm^−1^ was due to the C=O stretching of lignin. The peak at 1423 cm^−1^ shows the CH_2_ stretching of cellulose, and the peak at 1240 cm^−1^ shows the C=O stretching of hemicellulose [64,69]. The prominent peak at 1026 cm^−1^ was caused by a lignin C-OH stretching [65].

### 3.5. XPS Analysis of NRL Fiber

XPS analysis determined the chemical components on the surface of the NRL fiber and their compositions. The ratios of carbon/oxygen (C/O) and oxygen/carbon (O/C) can be seen in Table 4.

Carbon and oxygen concentrations of NRL fiber were determined as 68.37% and 18.33%, respectively. C/O and O/C ratios indicate the hydrophobic or hydrophilic character of the fiber surface [70]. The surfaces of cellulosic-based fibers contain oxygen and carbon. However, the different chemical contents of the fiber affect the O/C ratio [71].

C/O and O/C ratios of fibers were obtained using XPS data, and the hydrophilic or hydrophobic character of the NRL fiber surface was determined. The O/C ratio of NRL fiber (0.26) was lower than that of hemp (0.27), jute (0.46), kenaf (0.45), and sisal (0.29) fibers [49] and higher than that of flax (0.156) fibers [72], as previously shown in the literature. Generally, if the C/O ratio is high, the surface characteristic of fibers is hydrophobic; this is crucial for interfacial bonding when a cellulosic fiber is used as reinforcement in composite materials [73]. It was determined that the NRL fiber had a very high C/O ratio (3.72) and could have a potential for reinforcing composites.

**Figure 5:**
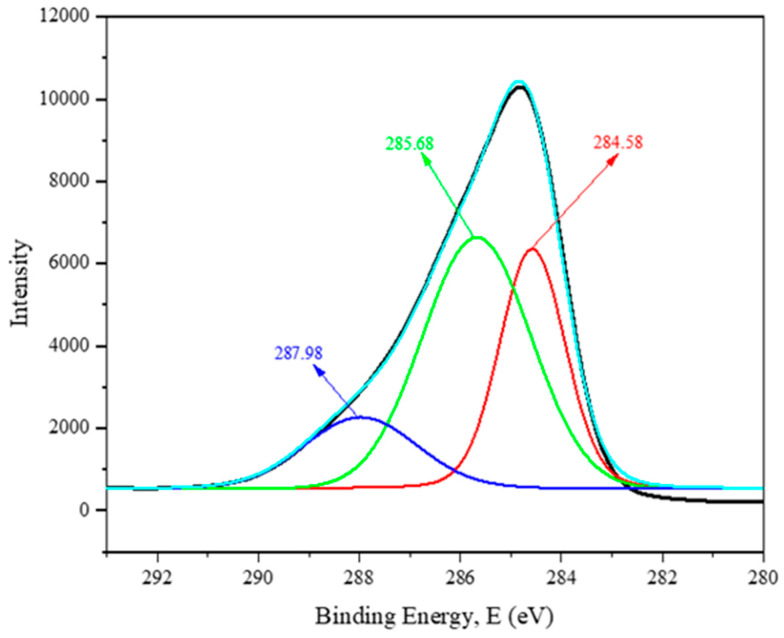
The XPS spectrum of NRL fiber.

Figure 5 shows the XPS spectrum curve of the NRL fiber. The peak at 284.58 eV (C-C/C-H) bonds corresponded to lignin and hemicellulose, and the peak at 285.68 eV may have indicated the presence of cellulose or ether [49]. The minor peak at 287.98 eV was related to the NRL fiber’s carbonyl groups (C=O/O-C-O).

### 3.6. XRD Analysis of NRL Fiber

**Figure 6:**
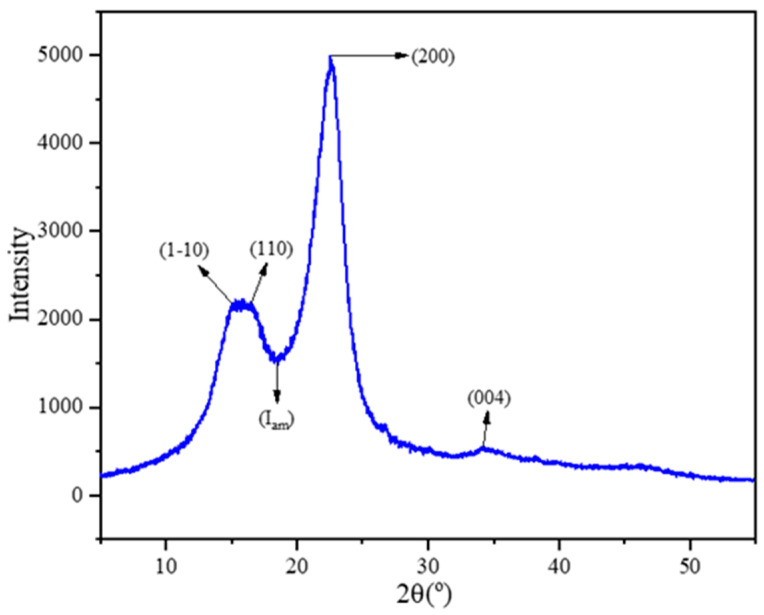
XRD pattern of NRL fiber.

The crystallinity index of NRL fiber was determined using X-ray diffraction analysis. The XRD pattern in Figure 6 shows two characteristic peaks attributed to cellulose I, the first one located at 2θ = 15.94°, where the two lattice planes that are (1 $\bar{1}$ 0/1 1 0) and (2 0 0) overlapped, and the second one was located at 2θ = 22.52° [71]. Moreover, the minimum peak between these two peaks was 2θ = 18.56° and corresponded to an amorphous cellulose field [74].

The CI value of the NRL fiber was 70% using the Segal formula [47]. It was similar to that of flax (70%), higher than that of jute (58.9%), Ramie (58%), and kenaf (61–69%), and lower than that of hemp (69–83%) and sisal (75%) bast fibers [75]. With the increase of crystalline regions in the fiber structure, cellulose chains can become more regular and increase the tensile properties applied to the fiber. Increasing the ratio of the crystalline region in cellulosic-based fibers positively affects the thermal strength of the fiber [76]. NRL fiber can be a good candidate for improving the mechanical properties of reinforced composites.

### 3.7. TGA Analysis of NRL Fiber

**Figure 7:**
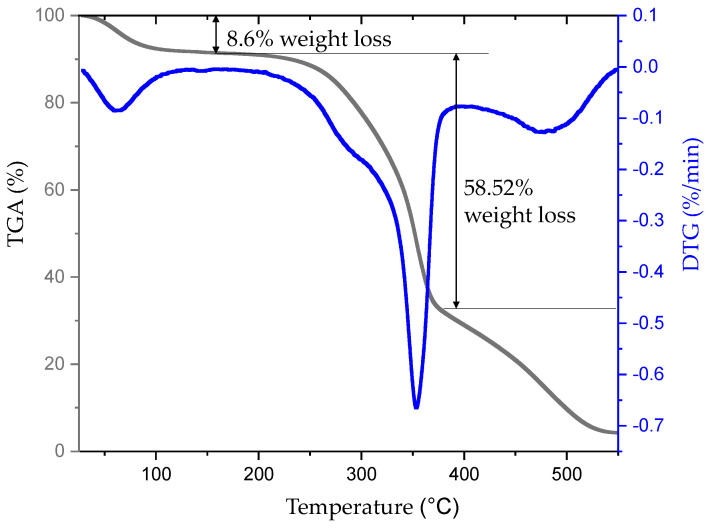
TGA/DTG curve of NRL fiber.

The curve obtained because of thermogravimetric analysis of NRL fiber is given in Figure 7. NRL fiber realized its first degradation between 25–100 °C with an 8.6% weight loss thanks to water hydrolysis. Also, its first degradation with the effect of heat is observed at 287.59 °C temperature, at which cellulose molecules begin to degrade (T_onset_) [77,78,79,80]. NRL fiber has a higher T_onset_ temperature and thermal stability than kenaf (219 °C), hemp (205 °C), jute (205 °C), bamboo (214), and bagasse (222) bast fibers [81]. This value indicates the fiber’s thermal stability and usability in the fiber-reinforced composites [79,82]. 

The NRL fiber’s maximum degradation temperature (total decomposition temperature of cellulose and lignin) (T_max_) was 353.90 °C with a 58.52% weight loss. It was higher than that of sisal (340 °C) and flax (345 °C) but lower than that of jute (365 °C) and kenaf (364 °C) [83,84] fibers stated in the literature. Due to its thermal stability at high temperatures can be an alternative reinforcement fiber for thermoplastic composites with relatively high-temperature values.

## 4. Conclusions

This study aimed to contribute to the search for alternative new raw materials to make sustainable, environmentally friendly, and petroleum-based products that textile and composite industries have demanded intensively in recent years. The water retting method was used to extract the fibers from the waste NRL plant stalks. The fibers’ chemical, physical, mechanical, and thermal properties were characterized. NRL plants are widely cultivated in the Adıyaman region of Turkey. After harvesting the leaves, the tobacco plant stalks are left in the fields or buried. Therefore, the potential for fiber production from these agro wastes is high in this region, and they are cultivated annually with a high production amount.

It was determined that NRL fibers have a higher cellulose content. The cellulosic and other chemical contents of the fiber were confirmed with FTIR analysis. The O/C ratios of the fiber surface were determined with XPS analysis, showing that the fiber was hydrophobic. The CI value was determined with XRD analysis, and mechanical properties (tensile strength, tensile modulus, and elongation) were examined with the single fiber tensile test. The thermal degradation values T_onset_ and T_max_ were obtained similarly to the other known cellulosic bast fibers. The characterization study of NRL fiber revealed that the fiber may have a potential for textile and composite applications. A blended yarn formed from NRL fiber and bast fibers can be investigated in future research.

## Figures and Tables

**Table 3:** Mechanical properties of NRL and other bast fibers.

Fiber	Tensile Strength (MPa)	Tensile Modulus (GPa)	Elongation (%)	Ref.
*Nicotiana rustica* L.	113.40	2.87	3.94	In this study
Cotton	400	12	3–10	[62]
Hemp	690	70	2–4	[42]
Flax	500–1500	27.6	2.7–3.2	[42]
Jute	93–773	26.5	1.5–1.8	[42]

**Table 4:** Surface chemical constituents of NRL fiber.

Fiber	Cls (%)	O1s (%)	N1s (%)	C/O (%)	O/C (%)
*Nicotiana rustica* L.	68.37	18.33	1.59	3.72	0.26

## Data Availability

Data are contained within the article.

## References

[1] Karabacak K. (2017). Tobacco Agriculture and Geographical Distribution in Turkey. CBD.

[2] Berlowitz I., García Torres E., Maake C., Wolf U., Martin-Soelch C. (2023). Indigenous-Amazonian Traditional Medicine’s Usage of the Tobacco Plant: A Transdisciplinary Ethnopsychological Mixed-Methods Case Study. Plants.

[3] Kishore K. (2014). Monograph of Tobacco (*Nicotiana tabacum*). Indian J. Drugs.

[4] Şahïn G., Taşligïl N. (2014). Türkiye’de Tütün (*Nicotiana tabacum* L.) Yetiştiriciliğinin Tarihsel Gelişimi ve Coğrafi Dağilimi. East. Geogr. Rev..

[5] (2013). Adıyaman Tobacco Report.

[6] Sifola M.I., Postiglione L. (2002). The Effect of Increasing NaCl in Irrigation Water on Growth, Gas Exchange and Yield of Tobacco Burley Type. Field Crops Res..

[7] Sifola M.I., del Piano L., Todisco D., Graziani G., Faugno S., Sannino M., Piscopo R., Salluzzo A., Cozzolino E. (2023). A Multipurpose Sustainable Farming System for Tobacco Crops in the Mediterranean Area. Sustainability.

[8] Tian W.-W., Xu F., Xing S.-J., Wu R., Yuan Z.-Y. (2023). Comprehensive Study on the Thermal Decomposition Process of Waste Tobacco Leaves and Stems to Investigate Their Bioenergy Potential: Kinetic, Thermodynamic, and Biochar Analysis. Thermochim. Acta.

[9] Kumar K., Goh K.M., Sparks D.L. (1999). Crop Residues and Management Practices: Effects on Soil Quality, Soil Nitrogen Dynamics, Crop Yield, and Nitrogen Recovery. Advances in Agronomy.

[10] Hirel B., Tétu T., Lea P.J., Dubois F. (2011). Improving Nitrogen Use Efficiency in Crops for Sustainable Agriculture. Sustainability.

[11] Zou X., Bk A., Abu-Izneid T., Aziz A., Devnath P., Rauf A., Mitra S., Emran T.B., Mujawah A.A.H., Lorenzo J.M. (2021). Current Advances of Functional Phytochemicals in *Nicotiana* Plant and Related Potential Value of Tobacco Processing Waste: A Review. Biomed. Pharmacother..

[12] Banožić M., Babić J., Jokić S. (2020). Recent Advances in Extraction of Bioactive Compounds from Tobacco Industrial Waste—a Review. Ind. Crops Prod..

[13] Sha Y., Yu H., Xiong J., Wang J., Fei T., Wu D., Yang K., Zhang L. (2023). Separation and Purification of Active Ingredients in Tobacco by Free-Flow Electrophoresis. Anal. Methods.

[14] Shi W., Li H., Zeng X., Zhang H., Qin X. (2019). The Extraction of Tobacco Protein from Discarded Tobacco Leaf by Hollow Fiber Membrane Integrated Process. Innov. Food Sci. Emerg. Technol..

[15] Muvhiiwa R., Mawere E., Moyo L.B., Tshuma L. (2021). Utilization of Cellulose in Tobacco (Nicotiana Tobacum) Stalks for Nitrocellulose Production. Heliyon.

[16] Ahmed M.J., Hameed B.H. (2024). Recent Progress on Tobacco Wastes–Derived Adsorbents for the Remediation of Aquatic Pollutants: A Review. Environ. Res..

[17] Gargol M., Klepka T., Klapiszewski Ł., Podkościelna B. (2021). Synthesis and Thermo-Mechanical Study of Epoxy Resin-Based Composites with Waste Fibers of Hemp as an Eco-Friendly Filler. Polymers.

[18] AL-Oqla F.M., Sapuan S.M., Ishak M.R., Nuraini A.A. (2015). Predicting the Potential of Agro Waste Fibers for Sustainable Automotive Industry Using a Decision Making Model. Comput. Electron. Agric..

[19] Kulić G., Radojičić V. (2011). Analysis of Cellulose Content in Stalks and Leaves of Large Leaf Tobacco. J. Agric. Sci..

[20] Tuzzin G., Godinho M., Dettmer A., Zattera A.J. (2016). Nanofibrillated Cellulose from Tobacco Industry Wastes. Carbohydr. Polym..

[21] Lavanya D., Kulkarni P.K., Dixit M., Raavi P.K., Krishna L.N.V. (2011). Sources of Cellulose and Their Applications—A Review. Int. J. Drug Formul. Res..

[22] Hinterstoisser B., Salmén L. (2000). Application of Dynamic 2D FTIR to Cellulose. Vib. Spectrosc..

[23] Bochek A.M. (2003). Effect of Hydrogen Bonding on Cellulose Solubility in Aqueous and Nonaqueous Solvents. Russ. J. Appl. Chem..

[24] Salem K.S., Kasera N.K., Rahman M.A., Jameel H., Habibi Y., Eichhorn S.J., French A.D., Pal L., Lucia L.A. (2023). Comparison and Assessment of Methods for Cellulose Crystallinity Determination. Chem. Soc. Rev..

[25] Felgueiras C., Azoia N.G., Gonçalves C., Gama M., Dourado F. (2021). Trends on the Cellulose-Based Textiles: Raw Materials and Technologies. Front. Bioeng. Biotechnol..

[26] Ramamoorthy S.K., Skrifvars M., Persson A. (2015). A Review of Natural Fibers Used in Biocomposites: Plant, Animal and Regenerated Cellulose Fibers. Polym. Rev..

[27] Rech F., da Silva F.P., Roldo L., Duarte L. (2023). Morphological, Chemical, And Thermal Characterization Of Tobacco Stalk For Application In Composite Materials. Eng. Technol. J..

[28] Ashori A., Ornelas M., Sheshmani S., Cordeiro N. (2012). Influence of Mild Alkaline Treatment on the Cellulosic Surfaces Active Sites. Carbohydr. Polym..

[29] Garcia K.R., Weiss-Angeli V., Koester L.S., dos Santos V., Brandalise R.N. (2021). Tobacco Stalk Lignocellulosic Nanofibers Characterization for Pharmaceutical Applications. Res. Soc. Dev..

[30] Çerçioğlu M. (2011). The Usage Possibility of Tobacco Waste in Sustainable Agriculture. J. Agric. Fac. Uludag Univ..

[31] Prima Indahsari O., Suryo Negoro A.H., Soeparjono S., Addy H.S. (2020). Erratum to: Contribution of Tobacco Waste for Agriculture. Proceedings of the E3S Web of Conferences.

[32] Popova V., Tumbarski Y., Ivanova T., Hadjikinova R., Stoyanova A. (2019). Tobacco Resinoid (*Nicotiana tabacum* L.) as an Active Ingredient of Cosmetic Gels. J. Appl. Pharm. Sci..

[33] Popova V., Ivanova T., Prokopov T., Nikolova M., Stoyanova A., Zheljazkov V.D. (2019). Carotenoid-Related Volatile Compounds of Tobacco (*Nicotiana Tabacum* L.) Essential Oils. Molecules.

[34] Fortunati E., Luzi F., Puglia D., Torre L., Puglia D., Fortunati E., Kenny J.M. (2016). Chapter 1—Extraction of Lignocellulosic Materials From Waste Products. Multifunctional Polymeric Nanocomposites Based on Cellulosic Reinforcements.

[35] Acda M.N., Cabangon R.J. (2013). Termite Resistance and Physico-Mechanical Properties of Particleboard Using Waste Tobacco Stalk and Wood Particles. Int. Biodeterior. Biodegrad..

[36] Wang S., Cheng Q. (2009). A Novel Process to Isolate Fibrils from Cellulose Fibers by High-Intensity Ultrasonication, Part 1: Process Optimization. J. Appl. Polym. Sci..

[37] Shadhin M., Rahman M., Jayaraman R., Chen Y., Mann D., Zhong W. (2023). Natural Biomass & Waste Biomass Fibers—Structures, Environmental Footprints, Sustainability, Degumming Methods, & Surface Modifications. Ind. Crops Prod..

[38] Tang Y., Yang S., Zhang N., Zhang J. (2014). Preparation and Characterization of Nanocrystalline Cellulose via Low-Intensity Ultrasonic-Assisted Sulfuric Acid Hydrolysis. Cellulose.

[39] Taherdanak M., Zilouei H. (2014). Improving Biogas Production from Wheat Plant Using Alkaline Pretreatment. Fuel.

[40] Satyamurthy P., Vigneshwaran N. (2013). A Novel Process for Synthesis of Spherical Nanocellulose by Controlled Hydrolysis of Microcrystalline Cellulose Using Anaerobic Microbial Consortium. Enzym. Microb. Technol..

[41] Pekpazar Y.K., Kilic U. (2020). Effects of Different Additives on Methane Productions and Feed Values of Tobacco Straws. Int. Multiling. J. Sci. Technol. (IMJST).

[42] Lee C.H., Khalina A., Lee S.H., Liu M. (2020). A Comprehensive Review on Bast Fibre Retting Process for Optimal Performance in Fibre-Reinforced Polymer Composites. Adv. Mater. Sci. Eng..

[43] Tahir P., Ahmed A.B., SaifulAzry S.O.A., Ahmed Z. (2011). Rettıng Process Of Some Bast Plant Fıbres And Its Effect On Fıbre Qualıty: A Revıew. BioResources.

[44] Duman M.N., Kocak E.D., Merdan N., Mistik I. (2017). Nonwoven Production from Agricultural Okra Wastes and Investigation of Their Thermal Conductivities. IOP Conf. Ser. Mater. Sci. Eng..

[45] (2018). Standard Test Methods for Density Determination of Flax Fiber.

[46] Mylsamy K., Rajendran I. (2010). Investigation on Physio-Chemical and Mechanical Properties of Raw and Alkali-Treated Agave Americana Fiber. J. Reinf. Plast. Compos..

[47] Segal L., Creely J.J., Martin A.E., Conrad C.M. (1959). An Empirical Method for Estimating the Degree of Crystallinity of Native Cellulose Using the X-ray Diffractometer. Text. Res. J..

[48] (2007). Standard Test Method for Tensile Properties of Single Textile Fibers.

[49] Dalmis R., Kilic G.B., Seki Y., Koktas S., Keskin O.Y. (2020). Characterization of a Novel Natural Cellulosic Fiber Extracted from the Stem of Chrysanthemum Morifolium. Cellulose.

[50] Shaker K., Waseem Ullah Khan R.M., Jabbar M., Umair M., Tariq A., Kashif M., Nawab Y. (2020). Extraction and Characterization of Novel Fibers from Vernonia Elaeagnifolia as a Potential Textile Fiber. Ind. Crops Prod..

[51] Ali A., Shaker K., Nawab Y., Jabbar M., Hussain T., Militky J., Baheti V. (2018). Hydrophobic Treatment of Natural Fibers and Their Composites—A Review. J. Ind. Text..

[52] Keskin O.Y., Dalmis R., Balci Kilic G., Seki Y., Koktas S. (2020). Extraction and Characterization of Cellulosic Fiber from Centaurea Solstitialis for Composites. Cellulose.

[53] Arthanarieswaran V.P., Kumaravel A., Saravanakumar S.S. (2015). Characterization of New Natural Cellulosic Fiber from Acacia Leucophloea Bark. Int. J. Polym. Anal. Charact..

[54] Rusdianto A.S., Amilia W., Sinta V.J.D. (2021). The Optimization Of Cellulose Content In Tobacco Stems (*Nicotiana Tabaccum* L.) With Acid Extraction Method And Alkaline Extraction Method. Int. J. Food Agric. Nat. Resour..

[55] Sun D., Sun S.-C., Wang B., Sun S.-F., Shi Q., Zheng L., Wang S.-F., Liu S.-J., Li M.-F., Cao X.-F. (2020). Effect of Various Pretreatments on Improving Cellulose Enzymatic Digestibility of Tobacco Stalk and the Structural Features of Co-Produced Hemicelluloses. Bioresour. Technol..

[56] Gedik G. (2021). Extraction of New Natural Cellulosic Fiber from *Trachelospermum jasminoides* (Star Jasmine) and Its Characterization for Textile and Composite Uses. Cellulose.

[57] Shakhes J., Marandi M.A.B., Zeinaly F., Saraian A., Saghafi T. (2011). Tobacco Residuals as Promising Lignocellulosic Materials for Pulp and Paper Industry. BioResources.

[58] Placet V., Day A., Beaugrand J. (2017). The Influence of Unintended Field Retting on the Physicochemical and Mechanical Properties of Industrial Hemp Bast Fibres. J. Mater. Sci..

[59] Liu Y., Ma Y., Yu J., Zhuang J., Wu S., Tong J. (2019). Development and Characterization of Alkali Treated Abaca Fiber Reinforced Friction Composites. Compos. Interfaces.

[60] Lampke A.B. (2005). Supriya Mishra, Thomas Plant Fibers as Reinforcement for Green Composites. Natural Fibers, Biopolymers, and Biocomposites.

[61] Hyness N.R.J., Vignesh N.J., Senthamaraikannan P., Saravanakumar S.S., Sanjay M.R. (2018). Characterization of New Natural Cellulosic Fiber from Heteropogon Contortus Plant. J. Nat. Fibers.

[62] Rajkumar R., Manikandan A., Saravanakumar S.S. (2016). Physicochemical Properties of Alkali-Treated New Cellulosic Fiber from Cotton Shell. Int. J. Polym. Anal. Charact..

[63] Dawit J.B., Regassa Y., Lemu H.G. (2020). Property Characterization of Acacia Tortilis for Natural Fiber Reinforced Polymer Composite. Results Mater..

[64] Belouadah Z., Ati A., Rokbi M. (2015). Characterization of New Natural Cellulosic Fiber from *Lygeum spartum* L. Carbohydr. Polym..

[65] Senthamaraikannan P., Kathiresan M. (2018). Characterization of Raw and Alkali Treated New Natural Cellulosic Fiber from *Coccinia grandis* L. Carbohydr. Polym..

[66] Liu D., Han G., Huang J., Zhang Y. (2009). Composition and Structure Study of Natural Nelumbo Nucifera Fiber. Carbohydr. Polym..

[67] Reddy K.O., Maheswari C.U., Shukla M., Rajulu A.V. (2012). Chemical Composition and Structural Characterization of Napier Grass Fibers. Mater. Lett..

[68] Jayaramudu J., Guduri B.R., Varada Rajulu A. (2010). Characterization of New Natural Cellulosic Fabric Grewia Tilifolia. Carbohydr. Polym..

[69] Saravanakumar S.S., Kumaravel A., Nagarajan T., Moorthy I.G. (2014). Investigation of Physico-Chemical Properties of Alkali-Treated *Prosopis Juliflora* Fibers. Int. J. Polym. Anal. Charact..

[70] Bulut Y., Aksit A. (2013). A Comparative Study on Chemical Treatment of Jute Fiber: Potassium Dichromate, Potassium Permanganate and Sodium Perborate Trihydrate. Cellulose.

[71] Manimaran P., Saravanan S.P., Prithiviraj M. (2021). Investigation of Physico Chemical Properties and Characterization of New Natural Cellulosic Fibers from the Bark of *Ficus Racemosa*. J. Nat. Fibers.

[72] Sgriccia N., Hawley M.C., Misra M. (2008). Characterization of Natural Fiber Surfaces and Natural Fiber Composites. Compos. Part A Appl. Sci. Manuf..

[73] Šernek M., Kamke F.A., Glasser W.G. (2004). Comparative Analysis of Inactivated Wood Surfaces. Holzforschung.

[74] French A.D., Santiago Cintrón M. (2013). Cellulose Polymorphy, Crystallite Size, and the Segal Crystallinity Index. Cellulose.

[75] Kılınç A.Ç., Köktaş S., Seki Y., Atagür M., Dalmış R., Erdoğan Ü.H., Göktaş A.A., Seydibeyoğlu M.Ö. (2018). Extraction and Investigation of Lightweight and Porous Natural Fiber from Conium Maculatum as a Potential Reinforcement for Composite Materials in Transportation. Compos. Part B Eng..

[76] Kim U.-J., Eom S.H., Wada M. (2010). Thermal Decomposition of Native Cellulose: Influence on Crystallite Size. Polym. Degrad. Stab..

[77] Diyana Z.N., Jumaidin R., Selamat M.Z., Alamjuri R.H., Md Yusof F.A. (2021). Extraction and Characterization of Natural Cellulosic Fiber from Pandanus Amaryllifolius Leaves. Polymers.

[78] Sheltami R.M., Abdullah I., Ahmad I., Dufresne A., Kargarzadeh H. (2012). Extraction of Cellulose Nanocrystals from Mengkuang Leaves (*Pandanus tectorius*). Carbohydr. Polym..

[79] Boumediri H., Bezazi A., Del Pino G.G., Haddad A., Scarpa F., Dufresne A. (2019). Extraction and Characterization of Vascular Bundle and Fiber Strand from Date Palm Rachis as Potential Bio-Reinforcement in Composite. Carbohydr. Polym..

[80] Ovalı S. (2023). Characterization of Lignocellulosic Glycyrrhiza Glabra Fibers as a Potential Reinforcement for Polymer Composites. J. Thermoplast. Compos. Mater..

[81] Yao F., Wu Q., Lei Y., Guo W., Xu Y. (2008). Thermal Decomposition Kinetics of Natural Fibers: Activation Energy with Dynamic Thermogravimetric Analysis. Polym. Degrad. Stab..

[82] Maache M., Bezazi A., Amroune S., Scarpa F., Dufresne A. (2017). Characterization of a Novel Natural Cellulosic Fiber from *Juncus effusus* L. Carbohydr. Polym..

[83] Manfredi L.B., Rodríguez E.S., Wladyka-Przybylak M., Vázquez A. (2006). Thermal Degradation and Fire Resistance of Unsaturated Polyester, Modified Acrylic Resins and Their Composites with Natural Fibres. Polym. Degrad. Stab..

[84] Kaya A.I. (2024). Extraction of Lightweight Platanus orientalis L. Fruit’s Stem Fiber and Determination of Its Mechanical and Physico-Chemical Properties and Potential of Its Use in Composites. Polymers.

